# Zinc–induced envelope stress diminishes type III secretion in enteropathogenic *Escherichia coli*

**DOI:** 10.1186/1471-2180-12-123

**Published:** 2012-06-24

**Authors:** Jay L Mellies, Katherine Thomas, Michael Turvey, Neil R Evans, John Crane, Ed Boedeker, Gregory C Benison

**Affiliations:** 1 Biology Department, Reed College, Portland OR, USA; 2 Division of Infectious Diseases, University at Buffalo, Buffalo, New York, USA; 3 Division of Gastroenterology, University of New Mexico, and the Albuquerque VA Medical Center, Albuquerque, New Mexico, USA

## Abstract

**Background:**

Dietary supplementation with zinc has been shown to reduce the duration and severity of diarrhoeal disease caused by Enteropathogenic *Escherichia coli*, common in infants in developing countries. Initially this therapeutic benefit was attributed to the correction of zinc deficiency in malnourished individuals, but recently evidence has emerged that zinc significantly impacts the pathogens themselves: zinc concentrations achievable by oral supplementation can reduce the expression of key virulence-related genes in EPEC and related organisms.

**Results:**

Here, we investigate three possible mechanisms for such zinc-induced changes in expression of EPEC virulence: direct interaction of zinc with regulators of LEE operons; genetic interaction of LEE operons with known regulators of zinc homeostasis; and finally, downregulation of LEE transcription associated with activation of the *σ*^E^envelope stress response by zinc. We find evidence only for the latter mechanism, including zinc-induced down-regulation of type III secretion in EPEC similar to that caused by ammonium metavanadate, another known inducer of the *σ*^E^stress response.

**Conclusions:**

We conclude therefore that envelope stress is a major mechanism by which zinc attenuates the virulence of EPEC and related pathogens.

## Background

Enteropathogenic *Escherichia coli* (EPEC) is a leading cause of infantile diarrhoea in developing countries [1]. Net secretory diarrhoea results from altered host cell signaling events, loosening of tight junctions and is exacerbated by the destruction of absorptive tissue, the host intestinal microvilli [2]. These phenotypes are mediated by a type III secretion system, a molecular syringe that secretes bacterial proteins into host cells, and is a common feature of many gram-negative pathogens [3]. The mechanism of EPEC diarrhoeal disease is similar to that of enterohemorrhagic *E. coli* (EHEC), and thus EPEC can be used as a surrogate for investigating disease caused by this more serious threat to public health. While, by definition, EPEC, possesses no diffusible toxins, EHEC in contrast produces Shiga toxin, causing bloody diarrhoea or hemorrhagic colitis. The production of Shiga toxin also can lead to the life-threatening complication, hemolytic uremic syndrome (HUS), which occurs in approximately 10% of reported cases of EHEC infection [4].

In developing countries, studies have shown that administering zinc to children with diarrhoea reduces the severity of disease [5,6]. It was initially hypothesized that this effect was due to correction of zinc deficiencies often seen in impoverished and malnourished children in these regions of the world. Certainly zinc is an important nutrient due to its fundamental role as a cofactor - over 300 zinc-depedent enzymes have been identified from all forms of life, with many of these such as carbonic anhydrase forming a basic part of human metabolism. Zinc is also found in non-enzymatic contexts in humans, for example its structural role in the ubiquitous zinc finger transcriptional regulators [7]. Zinc is also important for immune function, and zinc deficiency adversely affects the health and development of children [8,9]. However, a double-blind, randomized, controlled study involving 937 children with acute diarrhoea conducted in New Delhi, India demonstrated that zinc supplementation benefited children in the experimental group irrespective of the child’s initial plasma zinc level [5]. Thus beyond being an important co-factor necessary for immune and enzyme function in children, zinc also reduces the duration and severity of diarrhoeal disease caused by *E. coli*. For an initial study conducted in Calcutta, many, but not all of the reported cases were caused by EPEC. Thus some researchers have argued for greater use of zinc supplementation to treat bacterial diarrhoeal disease in children in the developing world [10].

In EPEC, zinc causes a reduction in net protein secretion via the type III secretion system [11], encoded within the pathogenicity island termed the locus of enterocyte effacement or LEE. As an underlying mechanism, this metal ion reduces the expression of *espA*, encoding the monomer of the molecular syringe, and *espB*, which in conjunction with *espD* encodes the pore that inserts into the membrane of the host cell [12]. The genes *espAespB* and *espD* are found within the *LEE4* operon of EPEC [13,14]. Evidence suggests that zinc dependent down regulation of *LEE4* involves the global regulator protein Ler, encoded within the *LEE1* operon. Zinc also reduces expression of *LEE1*, and thus Ler [11].

In our current study we sought to understand the underlying mechanism of how zinc reduces the expression of LEE genes of EPEC. We found no evidence to suggest that zinc directly acts on the regulatory protein Ler. Rather, we present evidence that zinc causes EPEC envelope stress, leading to a *σ*^E^-dependent stress response characterized by increased expression of *rpoE*. Treating EPEC with ammonium metavanadate (NH_4_VO_3_) – a known chemical inducer of the *σ*^E^-dependent response – caused a reduction in type III-dependent secretion similar to that observed in the presence of zinc. This is a first account of a specific mechanism on how zinc supplements reduce the duration and severity of disease caused by EPEC and related diarrhoeal pathogens.

## Results

### Millimolar concentrations of zinc are required to inhibit Ler binding

Previous studies indicated that exogenous zinc diminished EPEC pathogenesis, in part, by inhibiting expression of virulence genes. Specifically, expression of genes of the LEE, encoding components of the type III secretion system, were reduced in the presence of 0.1 to 0.5 mM zinc acetate [11,15]. Data suggested that, for the *LEE4* operon, encoding *espA*, zinc-dependent down-regulation required the global regulator Ler [14], which controls expression of the *LEE4* operon. Thus we initially posited that upon zinc stress cytoplasmic concentrations of this metal ion prevented Ler binding to *LEE4* regulatory DNA.

**Figure 1:**
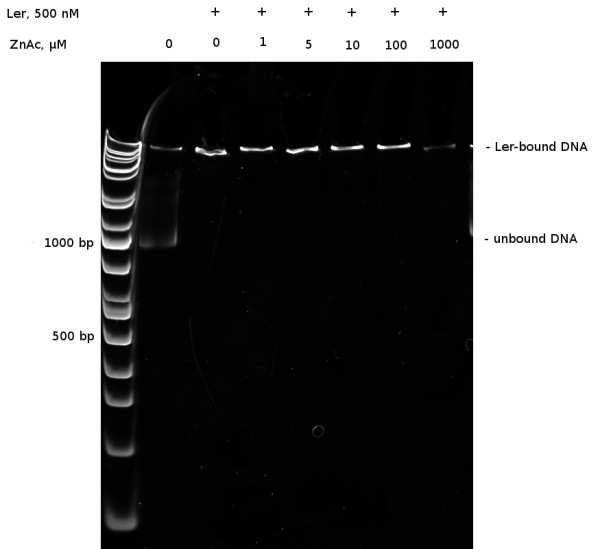
**Sub-millimolar zinc does not interfere with Ler binding to the *LEE4* operon***in vitro.* Ler binding to a fragment containing the *LEE4* promoter (bases -468 to +460 relative to the transcription start point) was assessed by EMSA in the presence of varied zinc acetate concentrations. Purified Ler protein at a final concentration of 0.5 *μ*M was incubated with 100 ng DNA at room temperature for 15 min, then separated on a non-denaturing 5% polyacrylamide gel by electrophoresis at 40V for 16 hours at 4°C. When Ler was present, essentially all of the DNA was bound in a nucleoprotein complex which was not disrupted by zinc acetate at any concentration up to 100 *μ*M, and only partially at 1000 *μ*M (the highest concentration tested). The upper and lower arrows mark the locations of bound and unbound DNA, respectively.

To test this hypothesis, we performed electrophoretic mobility shift assays (EMSA) using purified components (Figure 1). One hundred nanograms of *LEE4* regulatory DNA was incubated with 500 nM Ler protein with increasing amounts of zinc acetate. In the absence of added zinc, the Ler/DNA complex migrated poorly into the polyacrylamide gel compared to the DNA fragment alone, consistent with previously published data [16,17]. Concentrations of added zinc acetate up to 100 *μ*M showed no effect on the ability of Ler protein to bind and shift the *LEE4* regulatory DNA (Figure 1). At 1000 *μ*M, or 1 mM, zinc acetate we observed reduction in the ability of Ler to bind *LEE4* DNA by 80%. Thus *in vitro*, millimolar concentrations of zinc were necessary to disrupt Ler binding to regulatory DNA sequences.

Under normal physiological conditions, it is estimated that the concentration of free zinc within *E. coli* is in the femtomolar range, less then one zinc atom per cell [18], whereas the zinc quotient of the cell– that complexed with amino acids, ribosomal proteins and enzymes– reaches micromolar concentrations. Because millimolar concentrations of zinc acetate were necessary for disrupting Ler binding to *LEE4* (Figure 1) and no putative zinc binding domains are found within Ler (data not shown), we concluded that alterations of LEE gene expression by zinc did not involve direct interaction of zinc with the regulatory protein Ler.

### LEE gene expression is reduced by zinc in K-12 laboratory strains

**Figure 2:**
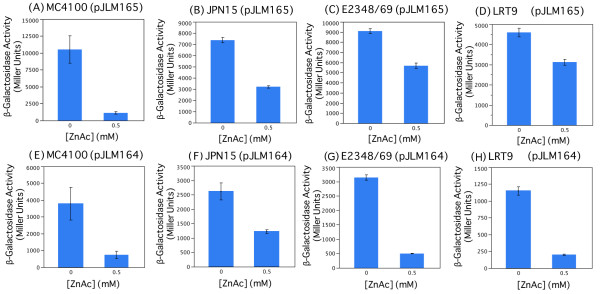
**Effect of zinc acetate on LEE gene expression.***β*-Galactosidase activities derived from plasmid pJLM165, *LEE4*-*lacZ*fusion in strains MC4100 (**A**), JPN15 (**B**), E2348/69 (**C**) and LRT9 (**D**); and plasmid pJLM164, *LEE1*-*lacZ*fusion in strains MC4100 (**E**), JPN15 (**F**), E2348/69 (**G**) and LRT9 (**H**) were monitored in the presence and absence of zinc in the tissue culture medium DMEM. Overnight cultures grown in LB were inoculated by 1:100 dilution into DMEM buffered with 25 mM HEPES, pH 7.4, and 50 *μ*g/ml kanamycin in the presence and absence of zinc acetate and harvested with OD_600_ of 0.3 to 0.5 in exponential growth. Activities were significantly greater in the 0 mM versus 0.5 mM zinc acetate conditions (A-H) for all cultures tested (Student’s t-test, p-value <0.05).

To further our understanding of zinc alteration of LEE gene expression we transformed plasmids containing *LEE1*-*lacZ* and *LEE4*-*lacZ* fusions (pJLM164 and pJLM165; Table 1) into the prototypical EPEC strain E2348/69, EPEC strain LRT9, strain JPN15 lacking the EAF virulence plasmid, and the K-12 strain MC4100. Strains were grown in DMEM medium in the presence and absence of 0.5 mM zinc acetate, and assayed for *β*-galactosidase activity. *β*-galactosidase activity derived from the *LEE4* operon was significantly diminished in the presence of zinc in all four strains (Figures 2A-D). Similarly, *β*-galactosidase activity derived from the *LEE1*-*lacZ*, multi-copy fusion was also diminished by the presence of 0.5 mM zinc acetate in the four strains tested (Figures 2E-H).

**Table 1:** Bacterial strains and plasmids used in this study


**Strain or plasmid**	**Genotype or description**	**Source or reference**
Strains		
E2348/69	Prototype EPEC strain (serotype O127:H6)	[19]
JPN15	EAF plasmid-cured derivative of E2348/69	[20]
MC4100	*araD139Δ*(*argF*−*lac*)*U*169 *rpsL*150 *relA1flbB5301deoC1ptsF25rbsR*	[21]
JLM164	MC4100 *ΦLEE1*−*lacZ*	[14]
JLM165	MC4100 *ΦLEE4*−*lacZ*	[14]
SIP812	MC4100 *zur*::Spc^*r*^/Str^*r*^	[22]
TB742	MC4100 *ΔzntR*	[23]
CT32	MC4100 *rpoE*−*lacZ*	[24]
MCamp	MC4100 *bla*−*lacZ*	[25]
CVD452	E2348/69 *ΔescN*::*aphT*	[26]
LRT-9	EPEC O111:abH2	[27]
Plasmids		
pRS551	Promoterless *lacZ* reporter fusion vector	[28]
pVSAPR	*bla*−*lacZ*	[25]
pJLM164	*LEE 1*−*lacZ*	[14]
pJLM165	*LEE 4*−*lacZ*	[14]

As a control we determined whether 0.5 mM zinc acetate affected the growth rate of either EPEC or the laboratory strain MC4100. We found that the doubling times of EPEC strain E2348/69 were 93 and 104 minutes in DMEM for 0 or 0.5 mM zinc acetate added, whereas for MC4100 the doubling times were 41 and 77 minutes for 0 and 0.5 mM zinc acetate, respectively. Thus the growth rate of the pathogenic strain E2348/69 was slowed by ∼10% though that of the laboratory strain was more adversely affected by zinc. These results indicated that previous assays demonstrating zinc-mediated down-regulation of LEE genes using qRT-PCR [11,15] could be faithfully reproduced using a *lacZ* reporter gene system, that down-regulation of *LEE4* occurred in the absence of Ler in the K-12-derived strain MC4100, and because we could observe this regulation in MC4100 derivatives that the regulation was not specific to the EPEC pathotype.

### Down-regulation of LEE genes by zinc occurs in the absence of zinc ion homeostasis regulators Zur and ZntR

We took advantage of the fact that zinc down-regulation of LEE genes could be reconstituted in K-12-derived strains to determine whether the observed regulation involved regulators of zinc ion homeostasis. The Zur regulator represses expression of the *znuABC* zinc transporter when the bacterium has excess intracellular concentrations of zinc, while ZntR stimulates expression of the *zntA* exporter when excess concentrations of zinc are found within the cytoplasm [18,29]. In the MC4100 *Δzur* strain SIP812 containing the pJLM164 plasmid, *β*-galactosidase activity derived from the *LEE1* operon decreased from ∼5000 to 1000 Miller units in the presence of 0.3 mM zinc acetate, a 5-fold reduction (Student’s t-test; n=3;p< 0.05). Similarly, in the MC4100 *ΔzntR* strain containing the pJLM164 plasmid *β*-galactosidase activity decreased from ∼3500 to 500 Miller units, a 7-fold reduction (Student’s t-test; n=3;p< 0.05), in the presence of 0.3 mM zinc acetate. We therefore concluded that zinc-mediated repression of *LEE1*, encoding Ler, did not require the global regulators of zinc homeostasis Zur or ZntR.

### Zinc stress increases ** *rpoE* **expression

Previous publications have indicated that excess zinc induces the expression of genes involved in envelope stress [30,31]. While these studies were conducted in complex media, including LB, we wanted to determine if genes associated with envelope stress were stimulated by zinc in the tissue culture medium DMEM used in our assays. We therefore plated the MC4100-derived strains CT32, containing a single-copy *rpoE-lacZ* fusion, JLM164 and JLM165, containing *LEE1lacZ* and *LEE4lacZ* fusions, respectively, and as a negative control, strain MCamp containing a single-copy *bla-lacZ* fusion on DMEM agar. Sterile disks containing 15 *μ*l of varying concentrations of zinc acetate were placed on the lawns of bacteria on selective medium containing X-gal, and growth proceeded overnight at 37°C.

**Figure 3:**
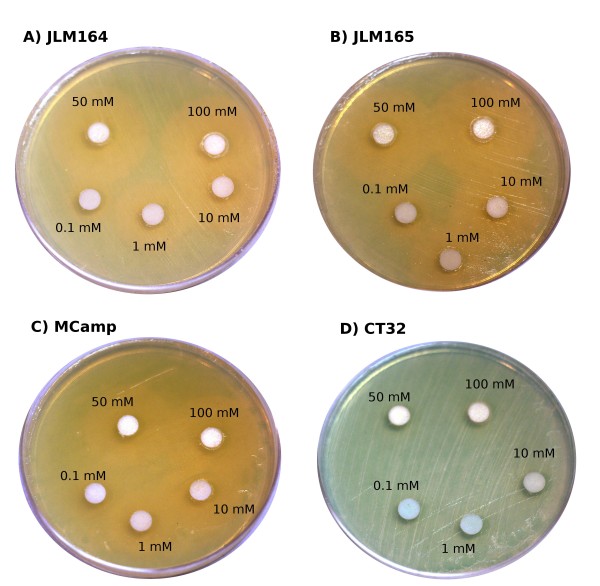
**Zinc downregulates both genes related and non-related to virulence but not *rpoE.* ** Overnight cultures of single-copy *lacZ* fusions JLM164 (*LEE*1−*lacZ*; **A**), JLM165 (*LEE*4−*lacZ*; **B**), MCamp (*bla*−*lacZ*; C), and CT32 (*rpoE*−*lacZ*; D) were spread evenly onto DMEM plates containing 30 mg/ml X-gal. Discs of sterile filter paper were dropped onto the lawn; 15 *μ*l of different concentrations of zinc acetate were placed on each disc (100 mM, 50 mM, 10 mM, 1 mM, 0.1 mM). These plates were grown for approximately 18 hours and then moved to 4°C for 6 hours to develop the blue color. Virulence genes were downregulated in the presence of zinc (A & B), but so was the *bla* gene encoding *β*-lactamase (**C**). In contrast, *rpoE* was not downregulated in the presence of zinc (**D**). Also of note is the small (∼1 mm) zone of growth inhibition around the 100 mM and 50 mM discs.

A relatively small zone of growth inhibition was noted surrounding the disk containing 100 mM zinc acetate for all strains tested (Figure 3). Thus high concentrations of zinc inhibited growth of these MC4100 derivatives. Consistent with our previous assays, we observed decreased *β*-galactosidase activities, indicated by a lack of blue color, surrounding the zinc acetate-containing disks on the plates containing the JLM164 and JLM165 strains, demonstrating that *LEE1* and *LEE4* expression was down-regulated in the presence of zinc acetate. However, we also observed similar down-regulation of *β*-galactosidase activity derived from the *bla-lacZ* negative control fusion from strain MCamp, suggesting that zinc caused a generalized down-regulation of gene expression in *E. coli*.

In contrast to these results, we did not observe a down-regulation of the *rpoE-lacZ* fusion from strain CT32 in the presence of any of the zinc acetate concentrations tested, indicated by blue color directly adjacent to the disks (Figure 3D). Consistent with this observation, by Miller assay [32], *β*-galactosidase activity derived from the *rpoE-lacZ* fusion strain CT32 in DMEM increased 1.7-fold from 512±24 to 865±19 Miller units (Student’s t-test; n=3;p< 0.05) in the presence of 0.3 mM zinc acetate. Because *rpoE* expression occurs via a mechanism whereby the alternate sigma factor *rpoE* is released from the cytoplasmic membrane upon insult [33], we concluded that *E. coli* grown in DMEM experiences envelope stress in the presence of zinc acetate, consistent with previously published reports using complex media [30,31].

### Zinc damages the EPEC envelope

**Figure 4:**
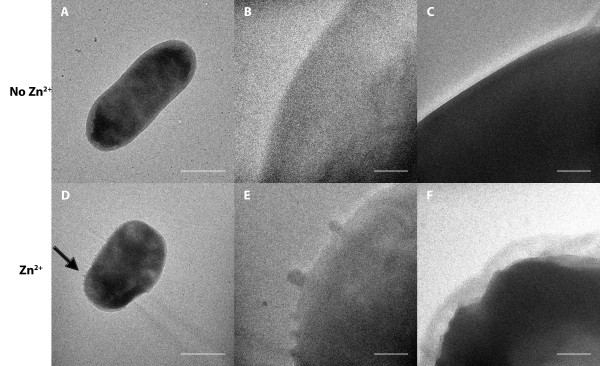
**The effects of zinc stress on the EPEC envelope imaged by transmission electron microscopy.** After 10-hour growth in DMEM medium, cultures were grown for an additional 5 hours in the absence **(A,B)** and presence **(D,E)** of 0.3 mM zinc acetate. EPEC bacteria were pelleted, the medium discarded, and bacteria then were resuspended in 0.1 M MgSO_4_. Samples were placed on carbon formvar grids, stained with 1.3% uranyl acetate and viewed by transmission electron microscopy. The same procedure was repeated with 1-hour growth in DMEM medium, followed by an additional 5-hours of growth in the absence **(C)** and presence **(F)** of 0.3 mM zinc acetate. Arrow points to outer membrane blebs in **(D)**. **(A,D)** Bars 1.0 *μ*m; **(B-C,D-F)** Bars 0.1 *μ*m.

To substantiate the damage to the EPEC envelope, comprising the inner membrane, periplasmic space, and outer membrane, suggested by our reporter gene fusion assays, we performed electron microscopy. EPEC bacteria were grown in DMEM tissue culture medium in the absence and presence of 0.3 mM zinc acetate. In the absence of zinc, the envelope of the bacteria appeared intact (Figures 4A-C). However, after growth in DMEM in the presence of zinc the outer membrane of the bacteria appeared compromised, and we observed what appeared to be multiple membrane blebs on individual bacteria (Figures 4D,E). Furthermore, we also observed bacteria with irregularly shaped inner membranes (Figure 4F). These data provided direct evidence that zinc damages the EPEC envelope.

### Chemical disruption of the EPEC envelope diminishes type III secretion

Zinc stimulates the expression of *rpoE* (Figure 3) and physically damages the EPEC envelope (Figure 4). These data demonstrated that, as for laboratory strains of *E. coli*, zinc causes envelope stress in EPEC. Along with down-regulation of LEE genes encoding type III secretion system components envelope stress could, at least in part, explain why zinc reduces diarrhoea in a rabbit illeal loop model of infection [11]. To test this hypothesis we monitored proteins secreted from EPEC grown in DMEM in the presence of ammonium metavanadate (NH_4_VO_3_). Ammonium metavanadate causes envelope stress and specifically stimulates the *rpoE* regulon [24,34]. Thus our prediction was that this chemical, in a manner similar to zinc, would diminish protein secretion via the type III secretion system of EPEC strain E2348/69.

To test this prediction strain E2348/69 was grown in DMEM overnight, in static cultures in the presence of increasing concentrations of NH_4_VO_3_. Bacteria were pelleted, and secreted proteins were harvested from the supernatant by TCA-precipitation. To control for proteins being released from the bacteria independently from the type III secretion system, we also harvested supernatant proteins from the strain CVD452, deleted for *escN*, encoding the ATPase [26]. We monitored secretion in the presence of zinc because protein secretion was previously shown to be diminished in the presence of this metal ion [11].

**Figure 5:**
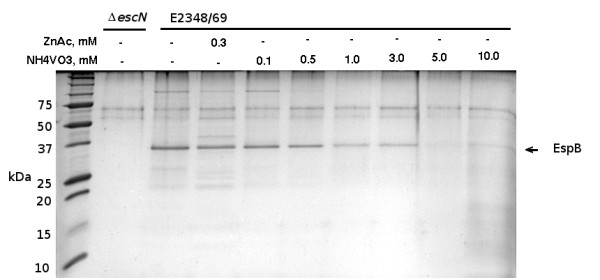
**Zinc and ammonium metavanadate both inhibit protein secretion from EPEC.** Cultures of EPEC strain E2348/69 were grown statically overnight in DMEM with varied concentrations of zinc acetate or ammonium metavanadate to an OD_600_ of 0.8 - 1.0. A culture of an EPEC strain deficient in type III secretion (*ΔescN*) was included as a control. Cells were removed by centrifugation, then proteins in the culture medium were precipitated with 25% trichloroacetic acid and visualized with SDS-PAGE. The volume of supernatant precipitated was chosen such that volume (ml)×culture OD_600_ = 6.0.

Upon separation by SDS-PAGE, for wild type strain E2348/69 we observed the EspB protein migrating to ∼33 kDa, which was dependent upon the type III system for secretion given that this band was absent in the lane corresponding to the *ΔescN*strain CVD452 (Figure 5; [35]). The identity of EspB was confirmed by an in-gel tryptic digest followed by mass spectrometry (data not shown). Increasing concentrations of NH_4_VO_3_ caused diminished protein secretion in a concentration dependent manner, such that at 10 mM of this chemical secretion of EspB was diminished by more than 70%. Because NH_4_VO_3_ stresses the bacterial envelope, specifically targeting the RpoE stress pathway, we concluded that stress to the EPEC envelope caused decreased protein secretion via the type III secretion system.

### Zinc precipitates phosphate from the tissue culture medium DMEM

**Figure 6:**
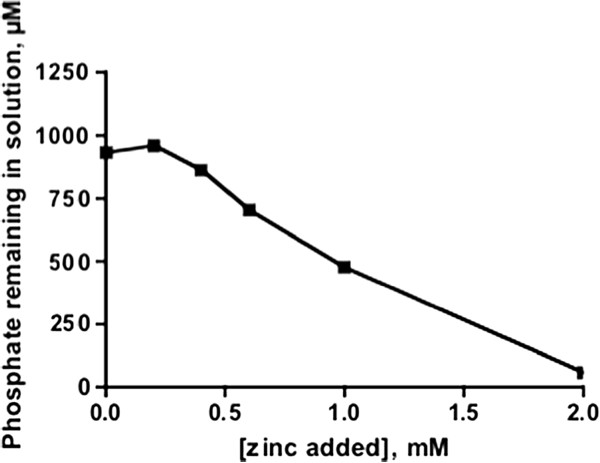
**Effect of added zinc on soluble phosphate remaining in DMEM.** Zinc acetate was added to DMEM and incubated at 37°C. Remaining soluble phosphate was quantitated with a Mol-Bio Green assay described in Methods.

Through the course of growing EPEC cultures in DMEM we observed that, not the doubling time, but rather the growth yield was modestly diminished in the presence of zinc acetate (data not shown). In addition, CFU/ml values after overnight growth in DMEM were ∼1.0 x 10^9^ versus 5.0 x 10^8^ in the absence and presence of 0.3 mM zinc. As phosphate is present in DMEM at 1 mM concentration, zinc phosphate is insoluble in solution, and we observed a small amount of white precipitate in DMEM in the presence of zinc acetate (data not shown), we hypothesized that the addition of zinc removed phosphate from this tissue culture medium. Indeed we observed that after the addition of millimolar concentrations of zinc, the concentration of soluble phosphate diminished in a linear fashion in DMEM (Figure 6). Therefore we concluded that zinc removed the essential element phosphorous from solution, and was the most likely explanation for the modestly diminished EPEC growth yield in the presence of zinc.

## Discussion

In this report we begin to elucidate the molecular mechanisms by which zinc diminishes EPEC virulence. Though previous data had indicated that zinc reduces LEE gene expression, in a Ler-dependent manner [11], as a negative control in this report we also observed that zinc reduced expression of the *bla* gene, encoding *β*-lactamase. Thus zinc appeared to be a general down regulator of a subset of *E. coli* genes [36], including those associated with EPEC virulence [11,15] (Figure 2). Consistent with this conclusion, we found no evidence for specific regulation by zinc interacting with Ler, or involvement of the major zinc homeostasis regulators Zur or ZntR. However, toward the goal of using dietary supplements to diminish the severity of disease caused by EPEC, and the related EHEC, zinc clearly reduces the expression of BFP, LEE genes, including the *LEE1* operon encoding Ler, and *stx* encoding the Shiga toxin [11,15] (Figure 2).

Looking for a general stress pathway to explain the observed down regulation of EPEC virulence genes, we observed stimulation of *rpoE* expression in the presence of zinc (Figure 3). We concluded that zinc caused envelope stress to EPEC grown in defined DMEM. Consistent with our observation, *rpoE* and a number of *rpoE*-dependent genes including *rpoH* and *htrA* were stimulated in the *E. coli* K-12 strain W3110 grown in LB in the presence of zinc chloride [31]. However, it is not likely that the RpoE sigma factor controls expression of LEE genes because the promoters identified for the LEE operons of EPEC were clearly RpoD-dependent, having consensus sequences highly similar to those of promoters transcribed by the *σ*^70^-containing RNA polymerase holoenzyme [14].

Zinc causes envelope stress, in part, by compromising protein tertiary structure, complexing with the thiol side chain of cysteine residues and/or disrupting disulfide bonds. Predictably, extracellular zinc causes a transient induction of the genes necessary for cysteine biosynthesis, thought to mop up excess cytoplasmic zinc [31]. A brief, transitory increase in intracellular zinc concentration most likely occurs inside of the bacterium, particularly for the strains containing mutations in either *zur* or *zntR*, upon addition of 0.3 to 0.5 mM zinc acetate to the culture medium. However, evidence suggests that zinc is quickly complexed to cysteine because the cysteine biosynthetic genes are stimulated by zinc stress [31] and then intracellular zinc concentrations return to normal conditions where free zinc is in the femtomolar range, less than one zinc molecule per bacterium [18].

In EPEC, the type III secretion system is assembled through the envelope, spanning the inner and outer membranes, and beyond, in order to inject effector proteins into the host cell cytoplasm [12,37,38]. Thus one would predict that zinc adversely affects the assembly, and integrity of the injectosome once assembled, ultimately preventing protein secretion. Here we demonstrate that zinc physically alters the EPEC envelope (Figure 4) and that the envelope stressor NH_4_VO_3_, which modifies lipid A of the LPS [34] and specifically stimulates the RpoE regulon, inhibits type III protein secretion in a manner similar to that observed for zinc [11] (Figure 5). Thus diminished LEE gene expression and disruption of type III system-dependent protein secretion via envelope stress most likely, largely explain reduced net secretory diarrhoea in the presence of zinc in an ileal loop model of infection using rabbit enteropathogenic *E. coli*[11,15].

The chemical environment within the ileal loops is likely to be altered by the presence of zinc. Notably, our results using the tissue culture medium DMEM (Figure 6) suggest that millimolar quantities of zinc within ileal loops will lead to the precipitation of zinc phosphate and thus reduced availability of phosphate, limiting the number of bacteria within the loops. Zinc acetate levels within the rabbit intestine reached 0.3 to 0.4 mM three days post administering of 10 mg of dietary zinc [15]. Thus this level of zinc within the rabbit intestine not only reduces virulence functions of the bacterium, but will also diminish the availability of phosphate. *E. coli* has two major inorganic phosphate transporters: the Pit system is a high velocity, low affinity system with a Km of 38.2 *μ*M, while the Pst system is a low velocity, high-affinity system having a Km of 0.4 *μ*M [39-41]. Therefore, in our experimentation (Figure 6), the level of phosphate did not reach levels low enough to inhibit growth, or reduce the doubling time, even in the presence of 1 mM zinc acetate, but some loss of the overall availability of phosphate in the DMEM resulted in the observed reduced growth yield.

## Conclusions

Zinc interacts with multiple entities in order to affect EPEC virulence- the host, the bacterium itself and the surrounding medium. In humans inadequate levels of dietary zinc lead to an imbalance of the Th1 and Th2 adaptive immune responses, in part by a loss in function of the zinc-containing, thymic hormone thymulin, necessary for T-cell maturation [42]. So certainly, malnourished children in developing countries experiencing zinc deficiencies will have impaired immune function. Previous reports clearly indicate that zinc reduces net secretory diarrhoea in a rabbit ileal loop model of infection [11,15], and our our data now establish that envelope stress and the resultant loss of type III secretion system function begin to explain results observed in the animal infection model. Furthermore, because zinc can be given in relatively large doses without toxicity, this metal ion might also act to remove phosphate from the intestinal lumen, limiting bacterial populations. In sum, our results argue for a more widespread use of dietary zinc supplements to reduce EPEC diarrhoea in children living in the developing regions of the world, but this therapy approach might also be effective against a number of related, type III secretion system containing Gram-negative, diarrhoeal pathogens, for which therapy options are becoming increasingly limited.

## Methods

### Bacterial strains and cultures

The bacterial strains used are listed in Table 1. Overnight cultures were grown at 37°C with aeration supplemented with the appropriate antibiotic at the following concentrations: kanamycin (50 *μ*g/ml), ampicillin (100 *μ*g/ml), chloramphenicol (30 *μ*g/ml), or streptomycin (50 *μ*g/ml). Overnight cultures were subcultured into Dulbecco’s modified Eagle medium (DMEM) at the dilutions indicated. DMEM in this report refers to DMEM-F12 (Sigma-Aldrich) containing L-glutamine and 4500 mg/L glucose, supplemented with 18 mM NaHCO_3_ and 25 mM HEPES, pH 7.4. DMEM-F12 from Sigma-Aldrich was previously determined to contain no more than 1.5 *μ*M zinc [15]. The heavy metal content of the HEPES used in all culture media was measured by the manufacturer (Promega) as less than 5 ppm. Therefore the media used in this study contain a negligible amount of zinc compared to the amounts added as a zinc acetate supplement (100 *μ*M or more).

### Electrophoretic Mobility Shift Assay (EMSA)

The *LEE4* regulatory fragment (bases -468 to +460 relative to the transription start point) was amplified with primers K1150 and K1153 (Table 2) by PCR using plasmid pJLM165 as template [14]. DNA fragments were separated by 1.0% agarose gel electrophoresis, stained with ethidium bromide, excised and purified using a QIAQuick Gel Extraction kit (Qiagen). Ler protein was expressed from a pBadMycHis vector and purified as described previously [17]. EMSA-based competition to assess Ler binding to *LEE4* regulatory DNA was performed by using non-denaturing 5% polyacrylamide gels. Polyacrylamide gels were prepared with a 37.5 : 1 acrylamide/bisacrylamide solution (Bio-Rad) following a standard protocol. Binding reaction mixtures containing 100 ng DNA, EMSA buffer (10 mM Tris, pH 7.4, 5 mM NaCl, 50 mM KCl, 50 mg/ml BSA), 0.5 *μ*M Ler, and zinc acetate at the indicated concentrations were incubated at room temperature for 15 min. After the addition of glycerol to a concentration of 2.5% (v/v), samples were separated by electrophoresis at 4°C overnight at 35 V. Gels were stained with ethidium bromide and imaged using a Bio-Rad Fluor-S MultiImager. Band intensities were quantified with the Gnu Image Manipulation Program (http://www.gimp.org/).

**Table 2:** Oligonucleotide primers used in this study


**Primer**	**Sequence 5’ - 3’**	**Strand**	**Target**	**Reference**
K1153	CCGGAATTCTGCCGATGGCACCAGACA	+	*LEE4*	[14]
K1150	CGCGGATCCTGCCAAACATCGCCAAAGTAG	−	*LEE4*	[14]

### ** *β* **-galactosidase assays

Plasmid pJLM164 containing a *LEE1lacZ* fusion, and plasmid pJLM165 containing a *LEE4lacZ*fusion were transformed into EPEC strains E2348/69 and LRT9, and into the plasmid-cured EPEC derivative JPN15 and the K-12 strain MC4100. Strains were cultured overnight in LB medium with 50 *μ*g/ml kanamycin and then subcultured 1:100 into 3 ml DMEM buffered with 25 mM HEPES, pH 7.4, in the presence and absence of 0.3 – 0.5 mM zinc acetate. Cells were harvested with OD_600_ between 0.3 and 0.5, and *β*-galactosidase activity was monitored by standard methods [32]. Three independent assays were performed from each culture.

### Disk diffusion assays

MC4100-derived strains JLM164, JLM165, MCamp, and CT32 containing single-copy *lacZ* reporter fusions were plated evenly on DMEM agar containing 30 *μ*g/ml X-gal. Sterile disks containing 15 *μ*l of varying concentrations of zinc acetate were placed on the plates which were then cultured overnight at 37°C. The plates were then moved to 4°C for 6 hours to develop the color and photographed.

### Transmission electron microscopy

Overnight cultures of EPEC bacteria grown in LB medium were diluted 1:100 into DMEM-F12 medium, described above. After 10 hours of incubation at 37°C with shaking, incubation was continued in the absence and presence of final concentration 0.3 mM zinc acetate. After 15 hours of growth, samples were pelleted by centrifugation, spent medium discarded, and resuspended in 100 *μ*l 0.1M MgSO_4_. Forty *μ*l samples were pipetted onto parafilm, and carbon-formvar grids, shiny side down, were placed on top of each drop and left for 7 minutes. The grid was then transferred to a 40-*μ*l drop of 1.3% uranyl acetate in water and left to stain for 40 seconds. The grid was then removed and blotted dry. Samples were viewed on a Philips CM120 transmission electron microscope. Negatively stained samples were viewed at ambient temperature. Images were recorded at 16-bit grayscale in Gatan digital micrograph 3 (DM3) format on a 1,024×1,024 pixel Gatan 794 charge-coupled device multiscan camera. Gatan digital micrograph 3.4.0 software was used to calculate power spectra and to convert DM3 format raw images and power spectra to eight-bit grayscale TIF images. Grids were imaged at the Oregon Health & Science University Electron Microscopy Core Facility.

### Secretion assays

EPEC strain E2348/69 was cultured overnight in 2 ml LB medium; the EPEC *escN* deletion strain CVD452 was similarly grown in LB containing 50 *μ*g/ml kanamycin. The cultures were diluted 100-fold into 20 ml DMEM containing 25 mM HEPES, pH 7.4, and various concentrations of zinc acetate or ammonium metavanadate in 125 ml flasks. Ammonium metavanadate was added from a 0.1 N stock solution prepared by adding the appropriate amount of solid NH_4_VO_3_ and raising the pH until complete dissolution was achieved; tests showed that adding NH_4_VO_3_ from this stock up to 2 mM did not change the pH of DMEM solutions appreciably. The cultures were grown statically overnight at 37°C, reaching an OD_600_ of 0.9 - 1.1. Cells were removed from the medium by centrifugation at 12,000 x g for 30 minutes. Secreted proteins remaining in the supernatant were precipitated by adding 25% trichloroacetic acid to a volume of supernatant chosen such that volume (ml)×culture OD_600_ = 6.0. Precipitated proteins in this volume were pelleted by multiple spins into the same 1.5 ml collection tube at 12,000 g. Pellets were washed with acetone, dried at 95°C for 10 minutes, then resuspended by heating with Laemmli sample buffer (0.1 M Tris-Cl pH 6.8, 5% glycerol, 2% *β*-mercaptoethanol, 2% sodium docedcyl sulfate, 0.01% bromophenol blue) at 95°C for 10 minutes. Proteins were separated on 15% SDS-polyacrylamide gels and visualized by Coomassie blue staining. To verify the identity of the secreted protein, the band migrating at 33 kDa was cut out of the gel, and an in-gel tryptic digest was performed according to the manufacturer’s instructions (Promega; mass spectrometry grade Trypsin and Protease Max surfactant). MALDI-TOF mass spectrometry was used to confirm the presence of EspB tryptic fragments in the digest.

### Determination of phosphate in DMEM containing Zinc

DMEM was supplemented with 0 - 2.0 mM zinc acetate and allowed to incubate at 37°C for 2 hours. Insoluble material was removed by spin filtration, and then soluble phosphate was quantitated using a Bio-Mol Green assay (Enzo Life Sciences).

## Competing interests

The authors declare that they have no competing interests.

## Authors’ contributions

Experiments were performed by the following authors: EMSA assays - GCB and MT; Miller assays - GCB, JLM, KT, MT, and NRE; disc assays for gene expression and growth inhibition - MT and NRE; secretion assays - GCB; zinc precipitation measurements - JC; transmission electron microscopy - NRE. The manuscript was written primarily by JLM with review by all authors before submission. All authors read and approved the final manuscript.
